# MicroRNAs as Predictors of Lung-Cancer Resistance and Sensitivity to Cisplatin

**DOI:** 10.3390/ijms23147594

**Published:** 2022-07-08

**Authors:** Maria Konoshenko, Yuriy Lansukhay, Sergey Krasilnikov, Pavel Laktionov

**Affiliations:** 1 Institute of Chemical Biology and Fundamental Medicine, Siberian Branch, Russian Academy of Sciences, 630090 Novosibirsk, Russia; lakt@niboch.nsc.ru; 2 Meshalkin Siberian Federal Biomedical Research Center, Ministry of Public Health of the Russian Federation, 630055 Novosibirsk, Russia; lancuh1@mail.ru (Y.L.); professorkrasilnikov@rambler.ru (S.K.)

**Keywords:** lung cancer, non-small cell lung cancer, cisplatin, DDP, chemotherapy, chemosensitivity, chemoresistance, therapeutic effectiveness markers, microRNA

## Abstract

Background: Platinum-based chemotherapy, cisplatin (DDP) specifically, is the main strategy for treating lung cancer (LC). However, currently, there is a lack of predictive drug-resistance markers, and there is increased interest in the development of a reliable and sensitive panels of markers for DDP chemotherapy-effectiveness prediction. MicroRNAs represent a perspective pool of markers for chemotherapy effectiveness. Objectives: Data on miRNAs associated with LC DDP chemotherapy response are summarized and analyzed. Materials and methods: A comprehensive review of the data in the literature and an analysis of bioinformatics resources were performed. The gene targets of miRNAs, as well as their reciprocal relationships with miRNAs, were studied using several databases. Results and Discussion: The complex analysis of bioinformatics resources and the literature indicated that the expressions of 12 miRNAs have a high predictive potential for LC DDP chemotherapy responses. The obtained information was discussed from the point of view of the main mechanisms of LC chemoresistance. Conclusions: An overview of the published data and bioinformatics resources, with respect to the predictive microRNA markers of chemotherapy response, is presented in this review. The selected microRNAs and gene panel have a high potential for predicting LC DDP sensitiveness or DDP resistance as well as for the development of a DDP co-therapy.

## 1. Introduction

According to the GLOBOCAN data, lung cancer (LC) maintains its leading position and ranks first among men in both morbidity and mortality [1]. In 2020, 2.2 million new cases of lung cancer and 1.8 million deaths were registered. According to the modern classification, there are two main types of lung cancer: non-small cell lung cancer (NSCLC) which occurs in approximately 85% of patients and small cell lung cancer, which occurs in approximately 15% of patients [2]. There are three main subtypes of NSCLCs: squamous cell carcinoma (25% of lung cancers), adenocarcinoma (40% of lung cancers), and large cell carcinoma (10% of lung cancers); the other types of NSCLCs include neuroendocrine tumors and carcinomas with pleomorphic, sarcomatoid, or sarcomatous elements [2]. Lung cancer is a severe disease that is difficult to treat. Currently, the most common treatment strategies for LC include surgery, chemotherapy, radiotherapy, and combinations of these treatments. The development of chemotherapy and radiotherapy resistance is a key issue in the progression of LC. As a result, in most countries, the five-year survival rate of patients with lung cancer is only about 10–20% [1].

## 2. Cisplatin and Lung Cancer Chemotherapy

The main method of LC treatment is chemotherapy. Platinum-based drugs are the gold-standard first-line treatments (ASCO and NCCN). The use of platinum-based drugs (cisplatin and carboplatin), including in combination with other chemotherapy agents (taxanes, pemetrexed, and antimetabolites), can achieve an overall survival for patients of 8 to 10 months on average [3]. A large and randomized comparison of four co-therapy regimens for stage IIIb and IV LC (cisplatin and paclitaxel, cisplatin and gemcitabine, cisplatin and docetaxel, and carboplatin and paclitaxel), which are the most commonly used regimens in clinical practice, showed that none of the used regimens had advantages over the others. The overall survival for all the studied regimens was about 10 months, with a one-year survival rate of 34% [4]. Patients with stage III LC undergo chemoradiotherapy (sequentially or simultaneously). This chemotherapy regimen is based on the basic drugs of the platinum group or their combination with other chemotherapy drugs, i.e., paclitaxel, etoposide, and vinblastine. The median progression-free survival of patients treated with chemoradiotherapy is poor (approximately eight months), with a five-year survival rate of only 15% [5]. An option to improve the situation is to switch to other treatment regimens, or example, treatment using the immunotherapy drug durvalumab, which is an inhibitor of the PDL 1 ligand that mobilizes the effector link of the antitumor immune system in a tumor microenvironment [6] or treatment of EGFR-positive LC patients using a combination of chemotherapy and tyrosine kinase inhibitors (platina-based chemotherapy and osemertinib) [7]. These chemotherapy schemes can achieve longer remission. However, these treatments have some limitations: immunotherapy is not available in all countries [1] and only about 15% of the general population of NSCLC patients have mutations in the EGFR gene [1,8].

Thus, platinum-based chemotherapy continues to be the main strategy for treating lung cancer [3,9]. Cisplatin (DDP) is the most widely used chemotherapy agent; it is an alkylating agent that effects inter- and intra-strand DNA cross-links, leading to cell-cycle arrest. However, drug resistance can develop, resulting in further development of a tumor and side effects such as myelosuppression, drug nephritis, nausea, vomiting, hearing loss, and polyneuropathy, which significantly reduce a patient’s quality of life [10]. Acquired chemoresistance during treatment is a major problem for clinicians and is a major cause of therapeutic failure [11]. Various mechanisms of tumor resistance to DDP have been described, and, most recently, these mechanisms have been classified as follows: (1) pre-target resistance (before cisplatin binds to DNA), (2) target resistance (directly associated with DNA-cisplatin adducts), (3) post-target resistance (associated with apoptosis caused by DDP-mediated DNA damage), and (4) off-target resistance (affecting molecular mechanisms that do not present obvious links to DDP-induced signals) [11]. Regardless of the resistance type, a tumor’s loss of sensitivity to DDP leaves a very short period of time for therapy correction aimed at increased patient survival. Clinical outcomes in the treatment of LC patients could be significantly improved through the introduction of non-invasive biomarker assays to predict and monitor the effectiveness of therapy [12]. However, there is a lack of reliable predictive drug-resistance markers and an urgent need to develop reproducible and highly sensitive panels of predictive markers for DDP-effectiveness assessment. Knowing a tumor’s response to cisplatin in advance would help clinicians, both before and during treatments, to select effective drugs and to adjust chemotherapy programs from one option to another in a timely manner. Efforts to identify such markers have primarily focused on the mechanisms underlying DDP resistance. DDP-resistance regulation represents a complicated network of many factors and signaling pathways. Obviously, a set of markers is needed to detect different types of tumors and, subsequently, to highlight the typical principal or driving aberrations specific to a particular tumor. MicroRNAs (miRNAs) could be promising candidate biomarkers for DDP resistance in LC, due to the multiple mechanisms by which they regulate the expression, and vice versa, for different target genes. Fortunately, there is considerable evidence on the association of aberrant miRNAs expression with DDP resistance in tumor cells (Tables S1 and S2). 

## 3. MicroRNAs and Lung Cancer Chemotherapy

Since miRNAs regulate of a wide spectrum of physiological and pathological processes in cells, they are secreted from cells and enter the extracellular medium and biological fluids [13]. MicroRNAs have been shown to be rather stable in biological fluids, including blood or bronchial lavage, in which they circulate in tight complexes with biopolymers or are packed in membrane-coated vesicles [14,15] for review. Cell-free miRNAs (cfmiRNAs) can be released from different tumor areas or tumor nodes, and, therefore, a cfmiRNA profile reflects a patient’s generalized tumor phenotype. Considering the well-developed protocols for cfmiRNA isolation and evaluation of their sets and concentrations, cfmiRNAs could be promising diagnostic markers [16]. The availability of liquid media, such as blood, sputum, and saliva, and methods that do not require invasive procedures have provided an opportunity for using liquid biopsies in the diagnosis of cancers, including LC [17]. The correlation between changes in miRNA expression and tumor development during treatment (aggressiveness and chemoresistance) have prompted the development of miRNA diagnostic panels and the emergence of prognostic and predictive markers for monitoring cancer as well as the development of new strategic solutions for the treatment of platinum-resistant LC ([18,19,20] for review). In fact, miRNA dysregulation in LC and under LC chemotherapy is involved in the regulation of the genes crucial to chemoresistance development: DNA repair, apoptosis, cell-cycle regulation, epithelial–mesenchymal transition (EMT), hypoxia, autophagy, drug efflux, cancer stem-cells activation (CSCs), etc. [20,21,22].

Numerous studies have aimed at identifying the miRNAs that mediate DDP response by investigating miRNAs that induce resistance/sensitivity to DDP in tumor cells or through comparative analyses of miRNA expressions in chemo-resistant and chemo-sensitive samples (cell lines and the tissues or biofluids of DDP-resistant and -sensitive LC patients). However, there are fewer studies that have aimed at exploring miRNAs differentially expressed under DDP chemotherapy.

In the present study, we aim to propose a preliminary panel of miRNAs for predicting the effectiveness of DDP chemotherapy via analysis of experimental data on the miRNAs’ involvement in DDP response and their cross analysis with the data of the bioinformatic resources that describe miRNAs and genes mediating DDP-response interconnection. Studies for analysis were selected from the PubMed and Science Direct databases based on the selection criteria described below.

Inclusion Criteria

Studies that described an association between miRNA expression and responses to DDP chemotherapy in lung adenocarcinoma and non-small cell lung cancer patients, including liquid biopsy studies as well as in models of cultivated cells and xenografts, were included for consideration.

The inclusion criteria were:

Studies that reported a change in miRNA expression in response to DDP chemotherapy in NSCLC;
Studies that reported the influence of miRNA expression on resistance/sensitivity to DDP;
Studies that aimed to compare miRNA expression in DDP-resistant and DDP-sensitive samples (tissues, serum of LC patients, cell lines, and xenograft models).

The exclusion criteria were:

- Studies published in languages other than English;
- Letters to the editor, case studies, or review articles;
- Bioinformatics data without experimental approval.

## 4. Comparison of miRNA Expression in DDP-Resistant and DDP-Sensitive Samples from LC Patients, Cell Lines, and Xenograft Models

Comparative studies of miRNA expression in therapy-resistant and -sensitive cancer cells of different origins represent a basic method of resistance markers’ identification. There are numerous such studies (Tables S1 and S2), however, only a few of these studies have used large-scale methods such as NGS. Most studies have analyzed the expression of a few miRNAs in LC DDP-sensitive and DDP-resistant cells and tumor-tissue samples using RT-PCR; the RT-PCR approach presents limited information on individual miRNA-expression differences (Tables S1 and S2), relationships with other miRNAs regulating the same gene targets or pathways, and interconnections of the pathways involved in response to DDP. In addition, there are only a few studies on cell-free miRNAs from blood plasma and miRNAs circulating in blood exosomes [23,24] or exosomes from a cell culture [25,26,27]. MicroRNAs packed in microvesicles, including exosomes, represent a convenient source of cell-free miRNAs for tumor diagnostics [28,29]. The miRNAs up- or downregulated in resistant samples represent perspective markers of resistance/sensitivity, correspondingly, however, they need to be additionally checked in this respect, for example, by directly studying their effect on DDP resistance by in vitro/ex vivo models.

## 5. Direct Effects of miRNA Overexpression/Depletion on DDP Sensitivity and Resistance

Changes in miRNA-expression levels in cells enable the direct evaluation of their effects on sensitivity/resistance to DPP chemotherapy. The results of studies of this type on sensitive and resistant cell lines and mice-xenograft models are presented in Tables S1 and S2. Parameters of chemosensitivity, such as the half-maximal inhibitory concentration (IC50), proliferation and apoptosis rates, cell-cycle arrest, cell viability, and migration and colony-formation ability, as well as tumor volume and weight, are usually assessed. Several miRNAs have been shown to be involved in DDP-resistance regulation using this approach, and many of these studies have included the study of the expression of the target genes that are crucial for a response to DDP (Tables S1 and S2) using luciferase reporter assay, qRT-PCR, and Western blot analysis. These investigations have helped to identify the network of miRNA–gene interactions involved in DDP-resistance development and to identify the principal miRNA players in the response to DPP. These miRNA and genes represent a valid set of preliminary prognostic markers.

## 6. Influence of DDP Chemotherapy on miRNA Expression

DDP chemotherapy, both as a powerful anticancer treatment and as a strong stressful intervention in organisms, causes significant changes in miRNA expression in tumor cells, as well as in many different normal cell types. However, until now, limited attention has been given to the analysis of the effect of DDP chemotherapy on the expression of miRNAs (Table 1).

Nevertheless, miRNAs deregulated by DDP therapy represent a pool of prospective biomarkers for the development of a co-therapy, because they may reflect the development of a secondary resistance to DDP, which, in turn, may be influenced by changes in miRNA-expression levels. Liquid-biopsy studies [26] are of special interest, because they allow continuous observation of changes in miRNA-expression levels during LC therapy. However, such studies are only at their starting point and require normal-tissue DDP-response filtering.

There are also some studies that have aimed to associate miRNA expression with simultaneous resistance/sensitivity to different chemotherapies, including DDP (Table 2). They include investigations of DDP and other platinum-based drugs, taxanes, cytostatic vincristine, and cetuximab (IgG1 against epidermal growth factor, Table 2). The results of such studies have indicated that miRNAs are involved in the regulation of drug resistance via both common and different drug mechanisms, including multidrug resistance.

## 7. Implication of miRNAs and Their Target Genes in Mechanisms of DDP LC Resistance

Well-known mechanisms of chemotherapy resistance (including lung-cancer DDP resistance) include apoptosis inhibition, cell-cycle progression, autophagy, drug transportation/detoxication (decreased drug uptake, activation of detoxification systems, and drug efflux), response to hypoxia, DNA repair, epithelial–mesenchymal transition (EMT), and cancer stem-cell (CSC) activation [42]. MicroRNAs have been shown to be involved in DDP-resistance development as well as to play an essential role in all the above-mentioned mechanisms.

**Figure 1:**
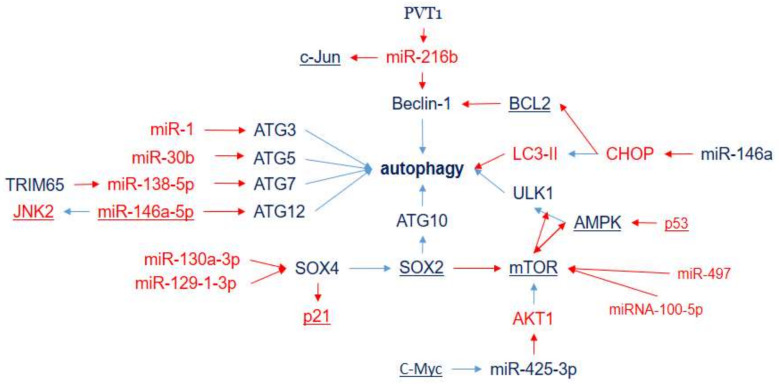
MicroRNAs and target genes implicated in LC DDP resistance via autophagy. Downregulation is shown by red arrows, and upregulation is shown by blue arrows. Overexpressed miRNAs and genes associated with DDP resistance (blue) and DDP sensitivity (red).

Autophagy implements the rearrangement of subcellular membranes for the subsequent autophagosome formation and lysosomal degradation of cytoplasmic contents and organelles [43]. Autophagy plays a dual suppressive or promoting role, which depends on the environmental context and the stage of tumorigenesis. During the early stages of cancer development, autophagy mainly acts as a survival pathway and a quality-control mechanism that may suppress cancer initiation and progression. At the late stage of cancer and under environmental stresses (such as hypoxia, starvation, hypoxia, heat stress, and accumulation of reactive oxygen species), autophagy promotes tumor survival, growth, and aggressiveness via metastasis [44]. Autophagy contributes to the response of cancer cells to chemotherapy agents: either as a protective mechanism for mediating resistance in response to chemotherapy or, in contrast, by inducing autophagic cell death, leading to sensitivity to chemotherapy [45]. However, when chemotherapy represents a strong stress, according to the published data (Tables S1 and S2), autophagy is mainly involved in DDP-resistance development. It has been shown that miRNAs, associated with the response to DDP therapy, are involved in the regulation of genes, which is crucial for all stages of autophagosome formation from initiation to maturation via autophagy-related genes (ATG), SOX4, SOX2, BCL2, Beclin-1, ULK, CHOP, etc. (Figure 1). Chemotherapy, both as a powerful anticancer treatment and as a strong stressful intervention in organisms, causes significant changes in miRNA expression in tumor cells as well as in many different normal cell types. However, until now, limited attention has been given to the analysis of the effect of DDP chemotherapy on the expression of miRNAs (Table 1).

For example, autophagy activating kinase 1 (ULK1) takes part in the serine/threonine protein kinase ULK1 complex, which is in the upstream position during phagophore assembly [46]. Beclin-1 (regulated by miR-216b) participates in the regulation of the formation of the autophagosomal membrane. Double-membrane autophagosomes are assembled under the control of the Atg12 (regulated by miR-146a), Atg5 (regulated by miR-30b), and Atg16 conjugation system and catalyzed by Atg7 (regulated by miR-138) and Atg10 (regulated by SOX2). LC3-phosphatidylethanolamine conjugate (LC3-II) is recruited to autophagosomal membranes. SOX2, influenced by SOX4 (regulated by miR-130a and miR-129), targets Atg10 to induce autophagy.

Although most of the interconnections (Figure 1) are clear and represent the summation of miRNA parallel and sequential effects, there are some uncertainties. On the one hand, for example, SOX2 initiates autophagy by repressing mTOR. Then, the repressed mTOR results in activation of the AMPK-ULK pathway. On the other hand, low expressions of miR-100-5p and miR-497 result in enhancement of mTOR expression associated with DDP-resistance development. The mechanistic target of rapamycin (mTOR, regulated by miR-100-5p and miR-497) plays the role of the core inhibitor of autophagy and has been shown to be regulated by miRNAs associated with DDP resistance. However, it seems that mTOR has dual action with respect to DDP resistance, since it activates DDP-resistance development via activation of cell proliferation and survival. It may reflect the dual role of autophagy in DDP resistance, as mentioned earlier. This example indicates the necessity for a complex analysis of miRNA-integrative effects that excludes ambiguous potential markers.

**Figure 2:**
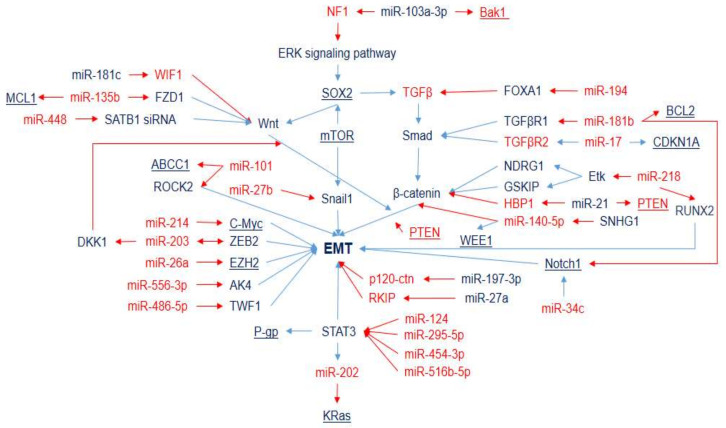
MicroRNAs and target genes implicated in LC DDP resistance via EMT. Downregulation is shown by red arrows and upregulation is shown by blue arrows. Overexpressed miRNAs and genes associated with DDP resistance (blue) and DDP sensitivity (red).

The epithelial–mesenchymal transition is an important step for cancer metastasis. It is a fundamental process in which epithelial cells gradually lose cell polarity and intercellular junctions, and acquire mesenchymal-cell phenotypic traits. Snail (regulated by miR-27b) is a significant EMT promoter in NSCLC [47]. The involvement of miRNAs in the DDP response via EMT regulation includes the ERK signaling pathway (miR-103a-3p), the PI3K/Akt signaling pathway, the TGF signaling pathway (miR-194, miR-181b, and miR-17), the Wnt/β-catenin signaling pathway (miR-181c, miR-135b, miR-448, miR-218, miR-21, and miR-140-5p), the Notch signaling pathway (miR-34c), the STAT signaling pathway (miR-125, miR-195-5p, miR-454-3p, and miR-516b-5p), and the enhancement of expression of EMT-related genes such as c-Myc (miR-214), ZEB2 (miR-203), EZH2 (miR-26a), AK4 (miR-556) TWF1 (miR-486-5p), and ROCK2 (miR-101) (Figure 2). EMT has also been associated with cancer stem cell (CSC) properties, for example, Myc (regulated by miR-214) mediates both cancer stem cells and EMT changes [48]. Moreover, there are genes (for example, BCL2 and c-Myc) that contribute to several key processes of DDP-resistance development simultaneously (they are presented in several figures and are highlighted by underlining). The miRNAs that affect such genes seem to have a synergetic effect and represent a very prospective pool of DDP-resistance markers.

**Figure 3:**
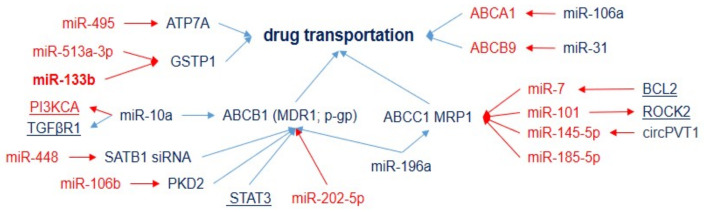
MicroRNAs inhibiting drug uptake or enhancing drug efflux and drug detoxification in DDP-resistant tumors. Downregulation is shown by red arrows, and upregulation is shown by blue arrows. MicroRNAs and genes associated with DDP resistance (blue) and DDP sensitivity (red).

The inhibition of drug uptake, enhancement of drug efflux, and detoxification are other key mediators of drug resistance. Transporters such as ATP7A and ATP7B contribute to the sequestration and efflux of platinum compounds that mediate resistance to DDP. According to the current data, miRNAs associated with DDP response act via several efflux-pump classes: the ATP-binding cassette (ABC) superfamily, multidrug-resistance protein (MDR, P-glycoprotein), and multidrug-resistance-associated proteins (MRPs, Figure 3).

For example, MDR1 (regulated by miR-448, miR-106b, miR-202-5, miR-196a, and by several miRNAs (miR-124, 295-5p, miR-454-3p, and miR-516b-5p) via STAT3) encodes a multidrug efflux pump that plays a crucial role in the development of resistance to a vast number of drugs, including platinum-based drugs and taxanes, and, thus, is considered to be a key molecular target for effectively attenuating drug resistance [49]. The elevation of copper-transporting P-type adenosine triphosphatases ATP7A (regulated by miR-495) has been associated with resistance to platinum drugs [50,51]; moreover, it has been identified as a negative prognostic factor for patients with NSCLC with platinum-based chemotherapy [51]. The expression of the ABC superfamily of transport proteins, such as MRP1/ABCC1 (regulated by miR-7, miR-101, miR-145-5p, miR-185-5p, and miR-196a), ABCCA1 (downregulated by miR-106a), and ABCB9 (downregulated by miR-31), also correlates with DDP and multidrug resistance (Tables S1 and S2). Other miRNAs (miR-133b and miR-513a) influence DDP resistance via glutathione S-transferase gene 1, a member of the family of dimeric phase II metabolic enzymes, which acts as a catalyst for the binding of intermediary metabolites to cofactors, transforming them into more hydrophilic molecules and, thus, facilitating their detoxification. GSTP1 has been shown to be associated with lung-cancer expansion; it may cause resistance by binding/inactivating cisplatin (platinum-glutathione conjugates), enhancing DNA repair, or reducing cisplatin-induced oxidative stress [52].

Cisplatin causes cytotoxicity by DNA damage with formation of cisplatin–DNA adducts. As a consequence, DNA-repair mechanisms are enhanced in DDP-resistant cells and, conversely, suppressed in DDP-sensitive cells. For example, miR-488 contributes to DDP resistance via suppression of eIF3a expression and, consequently, activation of XPC and RPA14 of the NER, which are involved in DNA-repair pathways in organisms including prevention of gene mutation and repair-DNA distortion [53]. The miR-92 family associated with DDP sensitivity downregulates RAD21, which is crucial for the homologous recombination of DNA during double-strand-break repair. DNA damage activates the stimulation of checkpoint mechanisms and leads to DNA repair and further cell-cycle progression or to cell death by apoptosis. Thus, DNA-damage-inducible transcript 3, induced in response to certain stressors, contributes to endoplasmic reticulum stress-induced apoptosis. However, under DDP therapy, miR-146a expression may be enhanced and lead to the suppression of CHOP and CHOP-induced apoptosis [54].

**Figure 4:**
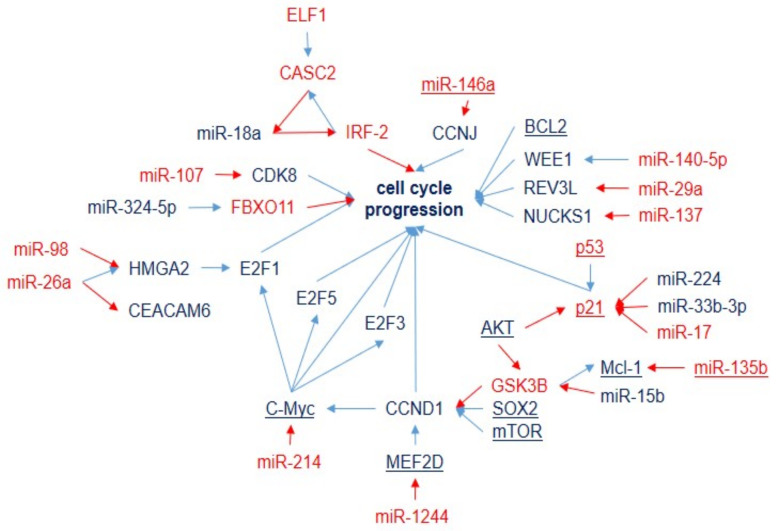
Cell-cycle regulation by miRNAs and target genes in LC-DDP-resistant tumors. Downregulation is shown by red arrows, and upregulation is shown by blue arrows. MicroRNAs and genes associated with DDP resistance (blue) and DDP sensitivity (red).

**Figure 5:**
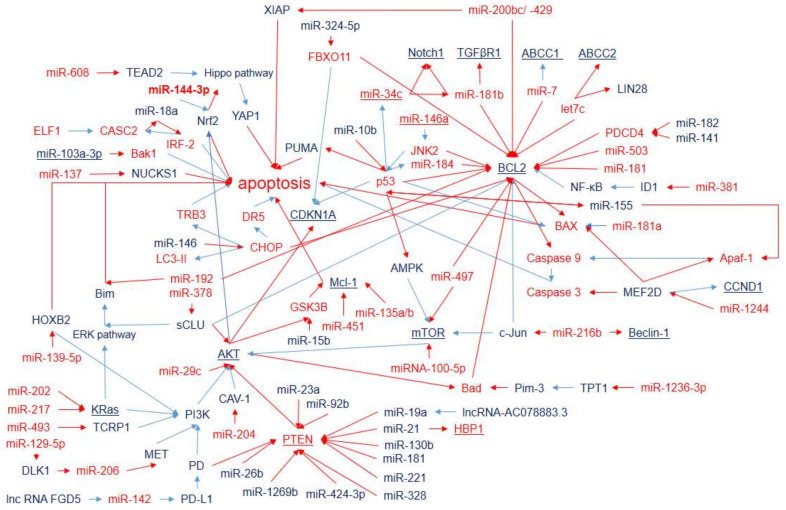
MicroRNAs and target genes regulating apoptosis in LC-DDP-resistant cells. Downregulation is shown by red arrows, and upregulation is shown by blue arrows. MicroRNAs and genes associated with DDP resistance (blue) and DDP sensitivity (red) are shown.

The miRNAs involved in DDP sensitivity/resistance via cell-cycle and apoptosis regulation are presented in Figure 4 and Figure 5. However, they are tightly related to each other and are separated artificially for easier identification.

Various studies have suggested that miRNAs are responsible for cell-cycle control by activating or inhibiting the expression of proteins involved in the response to DNA damage. Members of the BCL2 family have been the most frequently described target molecules among the different miRNAs associated with apoptosis related to DDP resistance in NSCLC cells (Table S2 and Figure 5). The complicated network of genes and miRNAs involved in the regulation of DDP resistance via BCL2 confirmed this. BCL2 is also considered to be an important anti-apoptotic protein that is involved in cisplatin-induced apoptosis (Figure 5). Other members of the BCL family, i.e., pro-apoptotic Bak1 and Bax, are regulated by miR-103a and miR-181a, correspondingly, and suppress their expression results in DDP resistance. Other widely represented mechanisms of miRNA influence on DDP resistance are the PTEN gene and the PI3K/Akt signaling pathway. This pathway is regulated by several miRNAs associated with DDP resistance (Figure 5) and influences apoptosis via NRF2 and BCL2 and the cell cycle via CDKN1.

The MiRNAs involved in CDDP-resistance of NSCLC cells, which are mainly assigned to apoptosis pathways, are summarized in Figure 5.

**Figure 6:**
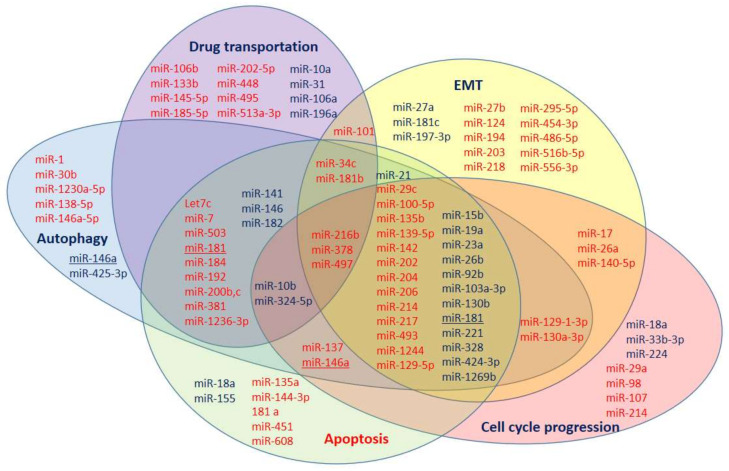
MicroRNA involvement in DDP-resistance development through EMT, drug transportation, apoptosis, cell cycle, and autophagy regulation. MicroRNAs, in which low expression is associated with DDP resistance development (red) and miRNAs, in which high expression is associated with DDP resistance development (blue).

The data on the involvement of miRNAs in processes of DDP resistance from Figure 1, Figure 2, Figure 3, Figure 4 and Figure 5 are summarized in Figure 6. Some miRNAs have multidirectional effects on DDP-resistance development (miR-146a and miR-181). Thus, they seem to be a doubtful choice as DDP-resistance markers. However, three of the analyzed miRNAs (miR-216b, miR-378, and miR-497) are involved in all the main processes (EMT, cell-cycle progression, drug transportation, apoptosis, and autophagy), and, therefore, have the highest potential as nonspecific DDP-resistance markers. Moreover, the distribution of miRNAs in Figure 6 evidence that all analyzed processes are crucial for DDP resistance and the development of a co-therapy for the DDP-resistance reversal should influence all of them.

## 8. Bioinformatics Analysis of miRNAs Involved in DDP-Response Regulation

MicroRNAs found in a few independent studies with different experimental designs are considered to be more valid as potential markers for the DDP response. To select such miRNAs, only miRNAs that were shown to be involved in the regulation of the ADT therapy response in three different types of experiments were considered for further analysis. The group of such miRNAs consisted of 9 miRNAs associated with DDP resistance and 23 miRNAs associated with DDP sensitivity (Table 3).

These miRNAs are involved in all key processes underlying PCA development: cell proliferation, epithelial–mesenchymal transition (EMT), apoptosis, cell-cycle progression, angiogenesis, metastasis, and invasion regulation (Table 4).

The Diana MirPath database presents processes regulated by all of these miRNAs. Twenty-four of the selected miRNAs, according to the Diana database, are involved in NSCL regulation (39 genes); there are eight miRNAs that are not involved: miR-146a, miR-200b, miR-203, miR-219a, miR-379, miR-381, miR-486, and miR-613. Moreover, all 32 selected miRNAs are involved in cell-cycle regulation (101 genes), the FoxO signaling pathway (95 genes), the Wnt signaling pathway (86 genes), the TNF signaling pathway (80 genes), the MAPK signaling pathway (156 genes), the TGF-beta signaling pathway (66 genes), the p53 signaling pathway (56 genes), the AMPK signaling pathway (89 genes), and the mTOR signaling pathway (46 genes).

**Figure 7:**
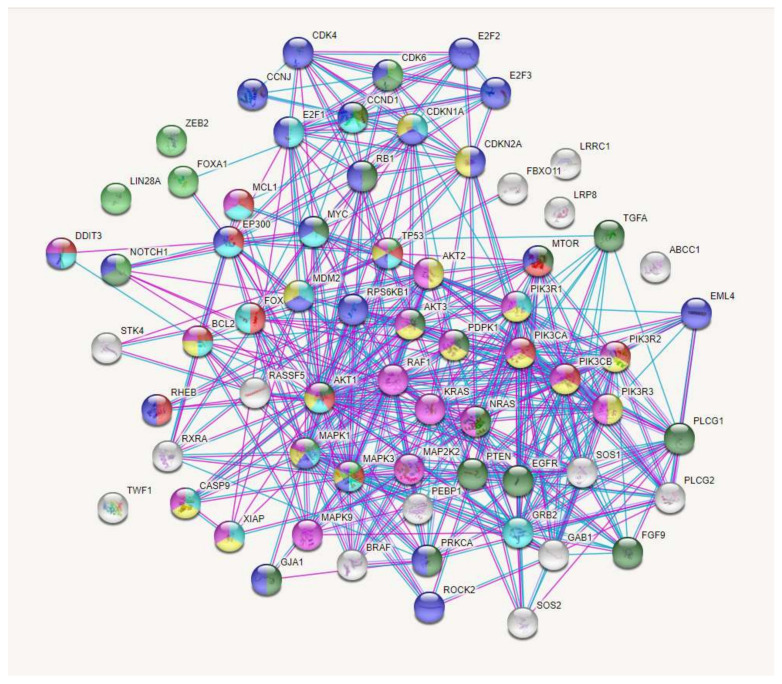
The interactions of proteins coded by genes that are regulated by miRNAs, which are the most valid as potential markers of DDP response and involved in lung-cancer regulation (STRING Database). Proteins involved in cellular response to DNA-damage stimulus (light blue); proteins involved in regulation of autophagy (red); proteins involved in stem-cell differentiation (green); proteins involved in regulation of epithelial-cell proliferation (dark green); proteins involved in cell-cycle regulation (blue); proteins involved in platinum drug resistance (yellow); proteins involved in apoptosis (pink).

The interactions of proteins encoded by the genes regulated by selected miRNAs and involved in LC regulation (58 genes) were analyzed using the STRING database (string-db. org). The complicated and closed network organized by these genes and the interactions between them are presented in Figure 7.

**Figure 8:**
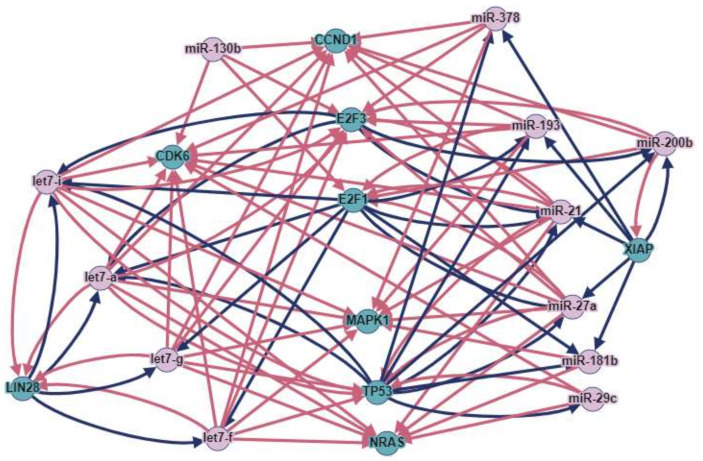
MicroRNAs and genes involved in DDP response, with the greatest number of interactions according to Diana and Targetscan databases. Red arrows represent downregulation, and blue arrows represent upregulation.

MicroRNAs and genes that are involved in a greater number of interactions represent the most robust potential markers of therapeutic effectiveness. To select such potential markers, we analyzed the regulation of genes by miRNAs and also whether transcription factors among selected genes could regulate miRNAs from the selected panel (using the TransmiR 2.0 database). The genes and miRNAs involved in the greatest number of interactions (more than 10) were then selected: let7a, i, g, f, miR-21, miR-27a, miR-29c, miR-130b, miR-181b, miR-193, miR-378, LIN28, CDK6, CCND1, E2F1, E2F3, MAPK1, TP53, NRAS, and XIAP (Figure 8).

To understand whether the 39 dysregulated miRNAs were specific for LC DDP chemotherapy or not, a further analysis using both DIANA and miRPath was performed. It was revealed that the miRNAs played significant roles in the development of oncological diseases. For example, all of the miRNAs were involved in the development of small cell lung cancer (63 genes), prostate cancer (68 genes), colorectal cancer (48 genes), renal cell carcinoma (58 genes), bladder cancer (29 genes), thyroid cancer (21 genes), endometrial cancer (39 genes), pancreatic cancer (51 genes), and glioma (47 genes). Previously, we analyzed the involvement of miRNAs in the regulation of chemotherapy resistance of prostate cancer [108]. Most of the studies analyzed in this article had been based on investigations of miRNAs in the regulation of responses to taxanes (docetaxel and paclitaxel). The combined analysis of bioinformatics resources and the available literature indicated that the expressions of eight microRNAs and nine genes were associated with chemotherapy response and had a high potential for the prediction of the prostate cancer chemotherapy response [108]. Interestingly, three miRNAs (miR-21, miR-27a, and miR-181b) and three genes (CCND1, E2F3, and TP53) were part of both combined miRNA panels, i.e., for prostate-cancer chemotherapy resistance and for lung-cancer DDP resistance. This indicates that these miRNAs and genes may represent a panel for nonspecific-cancer chemotherapy-resistance assessment. The miRNAs from the selected panel are involved in all crucial mechanisms of chemotherapy resistance, and, therefore, they may be non-specific for the type of the cancer and also for the type of the chemotherapy. This assumption still needs to be investigated. However, if the change in miRNA-expression level from the selected panel is not a diagnostic criterion, then the fact that they occur in other types of cancer becomes insignificant.

## 9. Conclusions

The data presented indicate that the selected miRNA and gene panel are strongly involved in DDP response and represent a perspective tool for the assessment and prediction of DDP therapy effectiveness. Nevertheless, their prognostic efficacy remains to be further confirmed by studies of randomized cohorts of patients.

## Figures and Tables

**Table 1:** The effect of DDP on miRNA level in LC samples.

No.	DDP R/DDP S miRNA	Downstream Regulated Target		In DDP R vs. DDP S Samples	Effect of DDP on miRNA Level	↑ of miRNA Expression → Chemoresistance	↓ of miRNA Expression → Chemosensitivity	Model: R/S Cells; Mice Xenografts	Reference
		Gene, Main Function/Pathway	Methods						
**1**	miR-33b-3p	P21	luciferase assay, RT-PCR, Western blot		↓ in cells	↑ cell viability, proliferation, promoted G1/S transition, DNA-damage response	↓ cell viability, G1 arrest,	S: A549 R: A549/DDP	[30]
**2**	miR-425-3p	AKT1, autophagy	luciferase assay, qRT-PCR, Western blot	↑ in cells, cells exosomes	↑ in serum exosomes, cell exosomes	↑ cell viability, ↓ apoptosis in S cells	↓ cell viability, ↑ apoptosis in R cells	S: A549 R: A549/DDP	[26]
**3**	miR-3195				↑ in cells, cell exosomes			S: A549	[25]
**4**	miR-3676-5p				↑ in cells, cell exosomes			S: A549	
**5**	miR-4443				↑ in cells, cell exosomes			S: A549	
**6**	Let7 (let-7a,-7b,-7c,-7d,-7e, -7f,-7g,-7i)	LIN28A,B	luciferase assay, IHC, RT-PCR, Western blot	↓ in tissues, cells	↓ in cells	↓ cell viability in R cells	↑ cell viability in S cells	S: A549 R: A549/DDP	[31]
**7**	miR-29c	AKT2	RT-PCR	↓ in tissues	↑ in cells	↓ cell viability in S ↓ tumor volume, proliferation (ki-67,AKT2) in X	↑ cell viability	S: SPC-A-1, A549 X: A549	[32]
**8**	miR-32	TRIM29		↓ in plasma	↑ in plasma				[24]
**9**	miR-181a				↑ in cells	↑ percentage of A549 cells with a G0-G1 DNA content ↑ proteolytic maturation of caspase-9 and caspase-3 triggered by CDDP ↑ proapoptotic member of the Bcl-2 family Bax	No effect	S: A549	[11]
**10**	miR-1244	Bax, MEF2D, cyclin D1, p53	qRT-PCR, Western blot		↓ in cells	↓ proliferation, ↑ apoptosis		S: A549, H522	[33]

DDP—cisplatin; DDP-S miRNA—miRNA associated with DDP sensitivity; DDP-R miRNA—miRNA associated with DDP resistance; R—chemotherapy-resistant cell line; S—chemotherapy-sensitive cell line; X—mice xenograft based on LC cell lines.

**Table 2:** miRNA and resistance to DDP and other chemotherapies.

No.	DDP R/DDP S miRNA	↑ of miRNA Expression	↓ of miRNA Expression	Model: (1) R/S Cells (2) Mice Xenografts	Drug	Reference
1	miR-181a	↑ migration, invasion, EMT in S	↓ migration, invasion, EMT in R cells	S: A549 R: A549/PTX, A549/DDP	DDP, paclitaxel	[34]
2	Let7f miR-29a	↓ cell viability in S, R		S: H2030 cells	DDP, carboplatin	[35]
3	miR-34c-3p	↓ cell viability, migration; ↑ apoptosis in cells ↓ tumor weight in X		S: A549, H1299 X: A549 mice	DDP, taxol	[36]
4	miR-137	↓ cell proliferation, migration, induced cell apoptosis, arrested cell cycle in G1 phase and reversed drug resistance in R cells; ↓ tumor volume, weight, VEGF (angiogenesis) in X	↑ cell growth, migration, cell survival, cell-cycle G1/S transition, resistance (CCK-8 assay) in S cells	S: A549 R: A549/CDDP X: A549/CDDP	DDP, paclitaxel	[37]
5	miR-200c	↓ cell viability, proliferation invasion, EMT; ↑ apoptosis		S: H1299, H596, and H522	DDP, cetuximab	[38]
6	miR-202	↓ cell viability, IC50; ↑ apoptosis in S; ↓ tumor volume in X		S: NCI-H441, A549 X: A549	DDP	[32]
		↓ IC50 in S			Oxaliplatin, carboplatin	
7	miR-216b	↓ IC50; ↓ tumor weight in X	↑ IC50	S: A549, PC9	DDP, carboplatin, oxaliplatin	[39]
8	miR-495	↓ cell viability, intracellular DDP accumulation in S, R	↑ cell viability	S: A549 R: A549/DDP	DDP, carboplatin, trans-/-diaminocyclohexaneoxalatoplatinum	[40]
9	miR-497	↓ cell viability, ↑ apoptosis in R	↑ cell viability in S	S: A549 R: A549/DDP	DDP, vincristine	[41]

DDP—cisplatin; DDP-S miRNA—miRNA associated with DDP sensitivity; DDP-R miRNA—miRNA associated with DDP resistance; R—chemotherapy-resistant cell line; S—chemotherapy-sensitive cell line; X—mice xenograft based on LC cell lines.

**Table 3:** miRNAs regulating DDP-therapy response, confirmed in three different types of experiments (data based on DDP-resistance studies and extracted from Tables S1 and S2).

DDP R/DDP S miRNA	Downstream Regulation	Reference
miR-21	PTEN	[55,56,57,58]
miR-27a	RKIP	[59,60]
miR-92b-3p	PTEN	[23,61]
miR-130b	PTEN	[62]
miR-146a	CHOP	[54]
miR-224	p-21	[62]
miR-324-5p	FBXO11	[63]
miR-425-3p	AKT1	[26]
miR-1269b	PTEN	[64]
let7 a,b,c,d,e,f,g,i	LIN28	[36,48]
miR-29c	AKT2	[65]
miR-30b-5p	LRP8	[66]
miR-34c-3p	Notch	[35]
miR-100-5p	mTOR	[25]
miR-101	ABCC1, ROCK2	[67,68]
miR-145-5p	ABCC1	[69]
miR-146a	JNK2, CEACAM6, CCNJ	[70,71,72]
miR-181b	BCL2, TGFβR1, Notch2	[73,74,75,76]
miR-193	LRRC1	[77]
miR-378	sCLU	[78]
miR-379	EIF4G2	[79]
miR-381	NFkB	[80]
miR-451a	MCL-1	[81,82,83]
miR-486-5p	TWF1	[84]
miR-613	GJA1	[85,86]

DDP—cisplatin; DDP-S miRNA—miRNA associated with DDP sensitivity; DDP-R miRNA—miRNA associated with DDP resistance.

**Table 4:** The involvement of selected miRNAs in crucial steps of tumorigenesis.

DDPR/DDP S miRNA	Apop-tosis	EMT	Cell Cycle Progression	Auto-Phagy	Proliferation	Cell Growth	Angio-Genesis	Meta-Stasis	Invasion	Reference
miR-21	↓				↑	↑	↑	↑	↑	[87,88]
miR-27a		↑								[60]
miR-130b	↑		↑		↑			↑	↑	[88,89,90]
Let7-a					↓			↓	↓	[91,92,93]
Let7-f										
Let7-g	↑		↓					↓		[94,95]
Let7-i				↓						[96]
miR-29c			↓							[65]
miR-181b	↑	↓			↓			↓		[73,76,97,98,99]
miR-193					↓			↓	↓	[100]
miR-200b	↑	↓			↓		↓	↓	↓	[101,102,103,104]
miR-378					↓	↓	↓	↓	↓	[105,106,107]

DDP—cisplatin; DDP-S miRNA—miRNA associated with DDP sensitivity; DDP-R miRNA—miRNA associated with DDP resistance; ↑ the increased expression of selected miRNA is associated with upregulation of process; ↓ the increased expression of selected miRNA is associated with downregulation of process.

## Supplementary Materials

The following supporting information can be downloaded at: https://www.mdpi.com/article/10.3390/ijms23147594/s1. References [25,109,110,111,112,113,114,115,116,117,118,119,120,121,122,123,124,125,126,127,128,129,130,131,132,133,134,135,136,137,138,139,140,141,142,143,144,145,146,147,148,149,150,151,152,153,154,155,156,157,158,159,160,161,162,163,164,165,166,167,168,169,170,171,172,173,174,175,176,177,178,179,180,181,182,183,184,185,186,187,188,189,190,191,192,193,194,195,196,197,198] are cited in the Supplementary Materials.
