# Supplementary material for: MicroRNAs as Predictors of Lung-Cancer Resistance and Sensitivity to Cisplatin
Source: Int J Mol Sci. 2022 Jul 8;23(14):7594. doi: 10.3390/ijms23147594 (PMC9321818; doi:10.3390/ijms23147594)
Supplement: Supplementary file 1 [file ijms-23-07594-s001.zip › ijms-1765855-supplementary.pdf]

Table S1. miRNAs associated with DDP resistance. Their downstream targets, expression in DDP-resistant vs DDP-sensitive samples, the effect of miRNA pressure on DDP-resistance parameters.

No.	DDP-R miRNA	Downstream regulated target		↑ in DDP resistant vs DDP sensitive samples	↑ of miRNA expression	↓ of miRNA expression	Model: resistant, sensitive cells; xenografts	Reference
		gene, main function/pathway)	methods					
1	miR-10a	PIK3CA, apoptosis	luciferase reporter assay, Western blot		↑ IC50 in S		S: A549, H1299	[109]
		MDR1, TGF βR1	ELISA assay	cells		↓ drug efflux, ↑ apoptosis in R	S: A549; R: A549/DDP	[110]
2	miR-10b	p-53, apoptosis	luciferase reporter assay, RT-PCR, Western blot		↑ proliferation, colony formation in S		S: A549	[111]
3	miR-15b	GSK-3β, apoptosis	luciferase reporter assay, RT-PCR, Western blot	cells	↑ cell viability, IC50 apoptosis in S	↓ cell viability, IC50 ↑ apoptosis in R	S: PC9-R, A549-R; R: PC9-R, A549-R	[112]
4	miR-18a	IRF-2	luciferase reporter assay, Western blot	tissues, cells			S: H226, A549; R: H226/DDP, A549/DDP	[113]
5	miR-19a	PTEN	luciferase reporter assay	cells			S: H460, A549; R: H460/DDP, A549/DDP	[114]
6	miR-21	PTEN	luciferase reporter assay, Western blot		↑ IC50, ↓ DDP-induced apoptosis in S	↓ IC50, ↑ apoptosis in S	S: A549	[55]
						↓ cell viability in S	S: H460, H157	[56]
					↑ cell viability, colony formation in R	↓ cell viability, colony formation, ↑ apoptosis in S, ↓ tumor volume, weight in X	S: A549; R: SK-MES-1; X: A549	[57]

		HBP1	luciferase reporter assay, Western blot		↑ migration, invasion, EMT in S	↓ migration, invasion, cell viability, EMT markers EMT in R	S: A549;<br>R: A549/DDP	[58]
7	miR-23a	PTEN, Akt	Western blot			↑ apoptosis in S	S: A549, NCI-H446	[115]
8	miR-26b	PTEN			↑ cell viability, migration in S		S: H1299, A549	[116]
9	miR-27a,b			cells			S: A549;<br>R: A549/DDP	[59]
10	miR-27a	RKIP, EMT	luciferase reporter assay, Western blot	tissues, cells	↑ IC50, proliferation, EMT in S, R, ↑ lung metastasis in XS	↓ IC50, proliferation, EMT in S, R, ↓ lung metastasis in XR	S: A549;<br>R: A549/DDP;<br>XS: A549;<br>XR: A549/DDP	[60]
11	miR-29			cells			S: A549;<br>R: A549/DDP	[59]
12	miR-31	ABCB9	luciferase reporter assay, RT-PCR and Western blot	cells	↑ cell viability, ↓ DDP-induced apoptosis in S, R	↓ cell viability, ↑ DDP-induced apoptosis in S, R	R: NCI-H1299;<br>S: SPC-A-1;<br>X: A549	[117]
				cells			S: A549;<br>R: A549/DDP	[59]
13	miR-33b-3p	P21	luciferase reporter assay, RT-PCR and Western blot		↑ cell viability, proliferation, promoted G1/S transition, DNA damage response	↓ cell viability, G1 arrest	S: A549;<br>R: A549/DDP	[30]
14	miR-34a			cells			S: A549;<br>R: A549/DDP	[118]
15	miR-92b	PTEN	luciferase reporter assay, RT-PCR, Western blot	cells	↑IC50 in S	↓ IC50, ↑ apoptosis in R	S: A549;<br>R: A549/CDDP	[61]
16	miR-92b-3p	PTEN, PTEN/AKT pathway	luciferase reporter assay, RT-PCR, Western blot	plasma exosomes, plasma	↑ IC50 in SBC-3, ↓ apoptosis in S, ↑ tumor volume, exosomal miR-92b-3p ↑ tumor volume in X	↑ apoptosis in SBC-3, ↓ tumor volume in X	S: SBC-3, SHP77;<br>X: SBC-3	[23]

17	miR-96	LMO7 (actin cytoskeleton)	luciferase reporter assay, Western blot			↓ cell viability, ↑apoptosis	S: A549	[119]
18	miR-98					↓ cell viability, ↑ apoptosis in S	S: A549, NCI-H460	[120]
19	miR-103a-3p	NF1 (ERK signaling pathway)		tissues, cells	↑ cell viability in S		S: A549, PC-9	[121]
				cells			S: A549;<br>R: A549/DDP	[59]
20	Exo-103a-3p	Bak1, apoptosis	luciferase reporter assay, RT-PCR		↑ cell viability ↓ apoptosis in S, ↑ tumor volume, ↓ apoptosis in X		S: H1650, H1299;<br>X: H1650	[122]
21	miR-106a	ABCA1	luciferase reporter assay, RT-PCR	cells	↑ cell viability in S	↓ cell viability in R	S: A549;<br>R: A549/DDP	[123]
22	miR-107			cells			S: A549;<br>R: A549/DDP	[59]
23	miR-130b	PTEN, Wnt/β-catenin	luciferase reporter assay, RT-PCR and Western blot	cells	↑ cell viability, ↓ apoptosis in S, ↑ tumor volume, ↓ apoptosis in X	↓ cell viability, ↑ apoptosis in R, S, ↓ tumor volume, ↑ apoptosis in X	S: A549, H446;<br>R: A549/CR, H446/CR;<br>X: A549	[124]
24	miR-141	PDCD4	luciferase reporter assay, RT-PCR and Western blot	cells		↓ cell viability, IC50, ↑ apoptosis in R	S: A549;<br>R: A549/DDP	[125]
25	miR-145	CDK6, cell cycle	Western blot, RT-PCR	cells			S: Calu6<br>R: Calu6 cisplatin-resistant sublines	[126]
26	miR-146a	CHOP, DNA reparation	luciferase reporter assay, RT-PCR Immunoblotting, Immunohistochemical staining of X tumor tissue	tissues, cells	↑ IC50 in A549, H446, ↑ tumor volume in X	reduction in cisplatin IC50 in A549/CDDP and H446/CDDP cells	S: A549, H446, H165;<br>R: A549/CDDP, H446/CDDP	[54]
27	miR-149			cells			S: A549;	[118]

							R: A549/DDP	
28	miR-155	TP53	luciferase reporter assay, Western blot		↑ proliferation, ↑ colony formation in S, ↑ proliferation, angiogenesis, ↓ apoptosis in X	↓ viability proliferation, angiogenesis, ↑ apoptosis in S, ↓ proliferation, angiogenesis, ↑ apoptosis ↓ number of tumors, tumor size and aggregate mass of metastases in X	S: A549;<br>X: A549-LVEV	[127]
		Apaf-1, apoptosis	RT-PCR, Western blot		↓ apoptosis in S		S: A549	[128]
29	miR-181a	PTEN	luciferase assay, Western blot	cells	↑ migration, invasion, EMT in S	↓ migration, invasion, EMT in R	S: A549;<br>R: A549/PTX, A549/DDP	[34]
				cells			S: A549;<br>R: A549/DDP	[118]
30	miR-181c	WIF1, Wnt/β-catenin pathway	luciferase assay, RT-PCR			↓ IC50, ↑ apoptosis in R,<br>↓ tumor volume in X	S: A549, H1299;<br>R: A549/CDDP, H1299/CDDP;<br>X: A549/CDDP	[129]
31	miR-182	PDCD4, apoptosis	RT-PCR, Western blot			↓ cell viability in S	S: A549	[130]
				cells			S: A549;<br>R: A549/DDP	[59]
32	miR-183			cells			S: A549;<br>R: A549/DDP	[59]
33	miR-192	Bim	luciferase assay, RT-PCR, Western blot		↑ IC50 value, ↓ DDP-induced apoptosis in S, R	↓ IC50 value, ↑ DDP-induced apoptosis in S, R	S: A549;<br>R: A549/DDP	[131]
34	miR-196a	MDR1, MRP1, ERCC1,	Western blot	Cells		↓ IC_{50} value, proliferation, colony formation,	S: A549;<br>R: A549/DDP	[132]

		survivin, and Bcl-2				↑ inhibitory rate, late-stage apoptosis in R		
35	miR-197			cells, cells exosomes			S: A549	[25]
36	miR-197-3p	p120 catenin, EMT	luciferase assay, qRT-PCR, Western blot		↑ viability and proliferation, ↓ apoptosis in S, ↑ tumor volume, weight in X	↓ viability and proliferation, ↑ apoptosis in S, ↓ tumor volume, weight in X	S: A549, H1299, H460, SPC-A-1; X: A549	[133]
37	miR-221	PTEN	qRT-PCR, Western blot	cells	↑ viability, proliferation in S, R	↓ viability, proliferation, ↑ cell senescence in S, R	S: A549; R: A549 /DDP	[134]
		PTEN	luciferase assay, Western blot	tissues	↑ colony formation, migration, invasion, ↓ cell arrest in G_0/G_1, ↓ apoptosis in R		R: SPC-A1/DDP, H520/DDP	[135]
				cells			S: A549; R: A549/DDP	[59]
38	miR-222			cells				
39	miR-224	p21$^{WAF1/CIP1}$, G(1)/S transition, apoptosis	luciferase assay, qRT-PCR, Western blot in LA cells, xenografts, qRT-PCR, immunostaining of LA tissues	tissues, cells	↑ colony formation, ↑IC_{50} value, ↓ cell arrest in G_0/G_1, ↓ DDP-induced apoptosis in S, ↑ tumor volume in X	↓ colony formation, ↓ IC_{50} value, ↑ cell arrest in G_0/G_1, ↑ DDP-induced apoptosis in R	R: A549/DDP, SPC-A1/DDP; S: A549, SPC-A1; X: A549	[62]
40	miR-324-5p	*FBXO11*, tumor suppressor, cell cycle progression, DNA replication, cell apoptosis	luciferase assay, qRT-PCR, Western blot	tissues, cells	↑ IC_{50} value, colony formation, ↓ apoptosis in S, ↑ tumor weight, proliferation, ↓ apoptosis in X	↓ IC_{50} value, colony formation, ↑ apoptosis in R	S: A549, SPC-A1; R: A549/CDDP, SPC-A1/CDDP; X: patient-derived xenografts	[63]

41	miR-328	PTEN	luciferase assay, qRT-PCR	tissues, cells		↓ proliferation, ↑ apoptosis in R	S: A549;<br>R: A549/DDP	[136]
42	miR-369-3p	SLC35F5	luciferase assay, qRT-PCR, Western blot	tissues	↑ proliferation, invasion, ↓ apoptosis in S	↓ proliferation, invasion; ↑ apoptosis in R	S: A549;<br>R: A549/DDP	[137]
43	miR-424-3p	PTEN	luciferase assay, Western blot			↓ cell viability, IC50, colony formation, ↑ apoptosis in S	S: A549, H460	[138]
44	miR-425-3p	AKT1, autophagy	luciferase assay, qRT-PCR, Western blot	cells, cells exosomes	↑ cell viability, ↓ apoptosis in S	↓ cell viability, ↑ apoptosis in R	S: A549;<br>R: A549/DDP	[26]
45	miRNA-488	eIF3a, NER signaling, DNA repair ability	luciferase reporter assay, qRT-PCR, Western blot	cells	↑ IC50, cell viability, ↓ apoptosis in S, R		S: A549;<br>R: A549/DDP	[53]
46	miR-630			cells	↑ cell viability, percentage of cells in G_0-G_1, ↓ DNA-damage response in S	no effect	S: A549	[139]
47	miR-638			cells			S: A549;<br>R: A549/DDP	[118]
48	miR-663			cells			S: A549;<br>R: A549/DDP	[118]
49	miR-642a-3p			cells, cells exosomes			S: A549	[25]
50	miR-1228			cells			S: A549;<br>R: A549/DDP	[118]
51	miR-1246			cells			S: A549;<br>R: A549/DDP	[118]
52	miR-1269b	PTEN, PI3K/AKT signaling pathway	luciferase assay, qRT-PCR, Western blot	tissues, cells	↑ IC50, cell viability proliferation, ↓ apoptosis in S, R, ↑ tumor growth in SX	↓ IC50, cell viability, proliferation, ↑ apoptosis in S, R, ↓ tumor growth in RX	S: A549;<br>R: A549/DDP;<br>SX: A549<br>RX: A549/DDP	[64]
53	miR-1469			cells			S: A549;<br>R: A549/DDP	[59]

54	miR-1470			cells			S: A549;<br>R: A549/DDP	[118]
55	miR-1908			cells			S: A549;<br>R: A549/DDP	[118]
56	miR-3172			cells			S: A549;<br>R: A549/DDP	[59]
57	miR-4281			cells			S: A549;<br>R: A549/DDP	[59]
58	miR-5100	RAB6, mitochondrial apoptosis	luciferase assay, qRT-PCR, Western blot	CSC cells	↑ IC50, CSC properties, sphere-formation ability, ↓ apoptosis in S	↓ IC50, CSC properties, ↑ apoptosis in S	S: A549, H1299 CD44+CD133+ A549, H1299	[140]
59	miR-297, let-7d-3p, miR-200b-3p, miR-3065-5p, miR-338-3p and miR-30e-3p			cell exosomes			S: A549	[27]

DDP–cisplatin; DDP-R miRNA–miRNA associated with DDP resistance; R–chemotherapy-resistant cell line; S–chemotherapy-sensitive cell line; X–mice xenograft based on LC cell lines

Table S2. miRNAs associated with DDP sensitivity: their downstream targets, miRNAs expression in DDP-resistant vs DDP-sensitive samples, the effect of miRNA pressure on DDP-resistance parameters.

No.	DDP-S miRNA	Negatively regulated target		↓ in DDP resistant vs DDP sensitive samples	↑ of miRNA expression	↓ of miRNA expression	Model: resistant, sensitive cells, xenografts	Reference
		gene, function/pathway	methods					
1	Let7 (let-7a, let-7b, let-7c, let-7d, let-7e, let-7f, let-7g, let-7i)	LIN28A, B	luciferase reporter assay, IHC, RT-PCR, Western blot	tissues, cells	↓ cell viability in R	↑ cell viability in S	S: A549<br>R: A549/DDP	[31]
2	Let7f miR-29a		luciferase reporter assay, Western blot		↓ cell viability in S		S: H2030	[36]
3	miR-let7i-5p			cells, cells exosomes			S: A549	[25]
4	miR-1	ATG3	luciferase reporter assay, Western blot	tissues, cells	↓ IC_{50}, autophagy, ↑ apoptosis in R		S: A549, H1299<br>R: A549/DDP, H1299/DDP	[141]
5	miR-7	MRP1/ABCC1, drug transportation	luciferase reporter assay, IHC, RT-PCR, Western blot	tissue				[142]
6	miR-15a-3p				↓ cell viability, ↑ autophagy, apoptosis in S, R		S: Calu1; R: CR-Calu1	[143]
7	miR-17 (miR-17, miR-20a, b, miR-93,	*CDKN1A*DNA synthesis	luciferase reporter assay, RT-PCR, Western blot	cells	↓ cell viability, ↑ apoptosis in R		S: A549;<br>R: A549/DDP	[59]

	miR-106a, b)							
8	miR-17	*TGFβR2*	luciferase reporter assay, RT-PCR, Western blot	cells	↓ cell viability, EMT, migration in R	↑ cell viability, EMT, migration in S	S: A549;<br>R: A549/DDP	[59]
9	miR20a/b							
10	miR-26a	CEACAM6, cell–cell interactions	luciferase reporter assay, Western blot	tissues, cells			S: A549;<br>R: A549/DDP	[71]
11	miR-27b-3p			cells, cells exosomes			S: A549	[25]
12	miR-27b	Snail, EMT	luciferase reporter assay, RT-PCR, Western blot		↓ proliferation, migration, EMT in S, ↓ tumor volume, weight in X		S: A549, H1299<br>X: H1299	[145]
				cells			S: A549;<br>R: A549/DDP	[118]
13	miR-29c	AKT2	RT-PCR	tissues	↓ cell viability in S, ↓ tumor volume, proliferation (ki-67 index and AKT2 in X	↑ cell viability in S	S: SPC-A-1, A549;<br>X: A549	[65]
14	miR-29a-3p			cells, exosomes			S: A549;<br>R: A549/DDP	[25]
15	miR-29a	REV3L	luciferase reporter assay, RT-PCR, Western blot		↓ viability in S cells, ↑ apoptosis	↑ viability in S cells	S: A549, H1650;<br>R: A549/DDP	[146]
16	miR-29a			cells			S: A549;<br>R: A549/DDP	[71]
17	miR-30b-5p	LRP8	luciferase reporter assay, RT-PCR, Western blot	cells	↓ viability, colony formation, migration, and invasion, ↑ apoptosis in S, ↓ tumor weight, volume in X	↑ viability, colony formation migration and invasion, ↓ apoptosis in S	S: A549, NCI-H1299;<br>R: A549/DDP;<br>X: A549 mice	[66]
18	miR-31			cells			S: A549;<br>R: A549/DDP	[71]
19	miR-32	TRIM29		plasma				[24]

20	miR-34a-5p		luciferase reporter assay, Western blot			↑ IC_{50}, proliferation, ↓ apoptosis	HCC827, H522	[147]
21	miR-34c-3p	NOTCH	luciferase reporter assay, RT-PCR, Western blot	tissues	↓ cell viability, migration, ↑ apoptosis in S, ↓ tumor weight in X		S: A549, H1299<br>X: A549 mice	[35]
22	miR-92 family (miR-92a,b; miR-25)	RAD21 the repair of DNA damage	luciferase reporter assay, RT-PCR, Western blot	cells	↓ cell viability, ↑ apoptosis in R		S: A549<br>R: A549/DDP	[148]
23	miR-92b			cells			S: A549<br>R: A549/DDP	[59]
24	miR-93			cells			S: A549<br>R: A549/DDP	[59]
25	miR-96-5p			cells			S: A549, H460;<br>R: H460-CisR, A549- CisR	[149]
26	miR-98	HMGA2			↓ cell viability, ↑ apoptosis in S, R		S: A549;<br>R: A549/DDP	[150]
27	miR-100-5p	mTOR	luciferase reporter assay, RT-PCR, Western blot	cells, cells exosomes	↓ IC_{50}, ↑ apoptosis in S, R, ↓ tumor volume, weight, ↑ apoptosis in X	↑ IC_{50}, ↓ apoptosis in S, R, ↑ tumor volume, weight, ↓ apoptosis in X	S: A549;<br>R: A549/DDP;<br>X: A549	[25]
28	miR-101	ABCC1	luciferase reporter assay, RT-PCR, Western blot		↓ cell viability, IC_{50}, colony formation, ↑ apoptosis in S	↑ cell viability, IC50, migration, invasion, colony formation in S	S: A549, H1299	[67]
		ROCK2, EMT	luciferase reporter assay, RT-PCR, Western blot	tissues, cells	↓ IC_{50}, migration, invasion, EMT, ↑ apoptosis in S, R		S: A549, NCI-H520;<br>R: A549-res, NCI-H520-res	[68]
29	miR-103a-3p	SOX4	luciferase reporter assay, RT-PCR, Western blot	cells	↓ IC_{50} in S, R	↑ IC_{50} in S, R	S: A549, H1299;<br>R: A549/DDP, H1299 / DDP	[151]

30	miR-106a			cells			S: A549; R: A549/DDP	[59]
31	miR-106b-5p	PKD2, drug transportation	luciferase reporter assay, Western blot	cells	↓ IC_{50}, invasion, ↑ apoptosis, G1 cell-cycle arrest in S, R		S: A549; R: A549/DDP	[152]
32	miR-107	CDK8	RT-PCR, Western blot		↓ cell viability in S		S: A549;	[153]
33	miR-124	STAT3	luciferase reporter assay, RT-PCR, Western blot	cells	↓ proliferation, migration, invasion, ↑ apoptosis in R		S: A549; R: A549/DDP	[154]
34	miR-125b-5p			cells, cells exosomes			S: A549; R: A549/DDP	[25]
35	miR-129-1-3p	SOX4	luciferase reporter assay, RT-PCR, Western blot		↓ IC_{50}, colony formation ↑ apoptosis in R cells	↑ IC_{50}, colony formation ↓apoptosis in R cells	R: H446/DDP, A549/DDP	[155]
36	miR-129-5p	DLK1	luciferase reporter assay, Western blot		↓ IC_{50}, colony formation ↑ apoptosis		S: A549, H460	[156]
37	miR-130a	SOX4	luciferase reporter assay, Western blot		↓ IC_{50}	↑ IC_{50}	S: A549, H1299; R: A549/DDP, H1299/DDP	[151]
38	miR-133	GSTP1	luciferase reporter assay, RT-PCR, Western blot	cells	↓ IC_{50}, survival, proliferation, colony formation, migration, ↑ apoptosis in S, R		S: A549, H1299; R: A549/DDP, H1299/DDP	[157]
39	135a/b	MCL1	luciferase reporter assay, Western blot	cells	↓ IC_{50} ↑ apoptosis in R	↑ IC_{50} in S	S: A549 R: A549/DDP	[158]
40	135b	FZD1	luciferase reporter assay, RT-PCR, Western blot	cells	↓ IC_{50}, proliferation ↑ apoptosis in R	↑ IC_{50}, proliferation, apoptosis in S	S: A549 R: A549/DDP	[159]
41	miR-137	NUCKS1, PI3K/AKT	luciferase reporter assay, immunoblotting, Western blot	tissues, cells	↓ cell proliferation, migration, induced cell apoptosis, arrest the cell cycle in G1 phase and reversed drug resistance in R, tumor volume and weight, ↓ VEGF (angiogenesis) in X	↑ cell growth, migration, cell survival, cell-cycle G1/S transition, resistance (CCK-8 assay) in S	S: A549, R: A549/CDDP X: A549/CDDP	[37]

42	miR-138-5p	ATG7, autophagy	luciferase reporter assay, RT-PCR,	tissues	↑ apoptosis in S, R		S: A549<br>R: A549/DDP	[160]
43	miR-138			cell exosomes	↓ cell viability in S, R		S: A549<br>R: A549/DDP	[161]
				cell			S: A549<br>R: A549/DDP	[118]
44	miR-139	HOXB2	luciferase reporter assay, RT-PCR, Western blot		↓ cell viability, ↑ apoptosis in S, R		A549	[161]
45	miR-140-5p	Wnt/β-catenin pathway	RT-PCR, Western blot		↓ cell proliferation, migration, invasion in R,<br>↓ tumor volume, weight in X		R: A549/DDP, H1299/DDP;<br>X: A549/DDP	[162]
46	miR-142	WEE1	luciferase reporter assay, RT-PCR		↓ proliferation, migration, invasion, and autophagy, ↑ apoptosis		R: A549/DDP, H1299/DDP	[163]
		Pd-L1, apoptosis	luciferase reporter assay, RT-PCR, Western blot	tissues, cells	↓ cell viability, migration, invasion in R		S: A549;<br>R: A549/DDP	[164]
47	miR-144	Nrf2	luciferase reporter assay, RT-PCR, Western blot	tissues	↓ cell viability	↑ cell viability	S: A549, H1299	[165]
48	miR-145-5p	ABCC1, drug transportation	luciferase reporter assay, RT-PCR, Western blot	tissues, cells	↓ IC_{50} in R		S: A549;<br>R: A549/DDR	[69]
49	miR-146a-5p	ATG12, autophagy	luciferase reporter assay, Western blot	cells, cell exosomes, serum exosomes	↓ cell viability in R	↑ cell viability in S	S: A549;<br>R: A549/DDR	[166]
50	miR-146a	JNK2, apoptosis	luciferase reporter assay	cells	↓ proliferation, invasion, Bcl-2, ↑ apoptosis, cell-cycle arrest in S, JNK2, P53 expression		S: A549;<br>R: A549/DDR	[70]
51	miR-148b	CEACAM6, cell–cell interactions	luciferase reporter assay, Western blot	tissues, cells			S: A549;<br>R: A549/DDR	[71]

		CCNJ, cell cycle	luciferase reporter assay, RT-PCR, Western blot		↓ cell viability, migration, invasion, ↑ cell-cycle arrest at G0/G1, apoptosis in R, ↓ tumor volume, ↑apoptosis in X		S: A549, SPC-A1 R: A549/DDP, SPC-A1/DDP; X: A549/DDP	[72]
		↑ DNMT1	luciferase reporter assay, RT-PCR, Western blot	cells	↓ cell viability, ↑ apoptosis in S, R		S: A549, SPC-A1; R: A549/DDP, SPC-A1/DDP	[167]
52	miR-181	PTEN, PTEN/ PI3K/AKT/mTOR pathway signaling	RT-PCR, Western blot		↓ proliferation, ↑ apoptosis, autophagy, PTEN/PI3K/AKT/mTOR pathway in R	↑ proliferation, ↓ apoptosis, autophagy, PTEN/PI3K/AKT/mTOR pathway in R	S: A549; R: A549/DDR	[75]
53	miR-181a				↑ percentage of cells in G0-G1, ↑ Bax in S	No effect	S: A549	[139]
54	miR-181b	BCL2 anti-apoptotic	luciferase reporter assay, Western blot	cells	↓ IC50 enhanced sensitivity to VCR, 5-Fu, CDDP, VP-16 and ADR, but not to MMC, ↑ apoptosis in S	↑ IC50 in R	S: A549; R: A549/CDDP	[73]
55	miR-96-5p, miR-182-5p, miR-183-5p	TGFβR1, Smad signaling pathway	luciferase reporter assay, RT-PCR, Western blot		↓ proliferation, migration, metastatic ability, EMT, cell viability, % cells in the S stage ↑ % cells in the G1 stage, apoptosis in S, R, ↓ tumor volume, lung metastasis formation, ↑ apoptosis in X	↑ cell viability, ↓ apoptosis in S, R	S: A549; R: A549/CDDP; X: A549/DDP nude mice	[74]
		Bcl-2,apoptosis	luciferase reporter assay, RT-PCR,		↓ proliferation, invasion		S: H446, H446R	[75]
		Notch2	luciferase reporter assay, Western blot		↓ cell viability, ↑ apoptosis in R, ↓ tumor volume, markers of cancer stemness in X	↑ cell viability, ↓ apoptosis in S	S: A549; R: A549/DDP; X: A549/DDP	[76]
		GLI2	luciferase assay, Western blot	cells	↓ proliferation in R		R: H460-CisR, A549- CisR	[149]

56	miR-183-5p			cells		↑ IC50, ↓ caspase3 in S, R	S: TL-1; R: TL-10	[168]
57	miR-184	Bcl-2, apoptosis	Western blot		↓ IC50, ↑ caspase3 in S, R			
58	miR-185-5p	ABCC1	luciferase reporter assay, RT-PCR, Western blot	cells	↓ proliferation, ↑ apoptosis in R		S: A549; R: A549/DDP	[169]
59	miR-193	LRRC1	luciferase reporter assay, RT-PCR, Western blot	tissues, cells	↓ proliferation, invasion, migration, colony formation in S, R, ↓ invasion, migration, ↑ apoptosis in X	↑proliferation, invasion, migration, colony formation	S: BEAS-2B, A549, H1299, H358, SPC-A-1; R: A549/DDP; X: A549, A549/DDP	[170]
60	miR-194			cells			S: A549; R: A549/DDP	[118]
61	miR-196a	FOXA1, EMT	luciferase reporter assay, Western blot	cells	↓ cell viability, proliferation, colony formation, migration, invasion, ↑ apoptosis, cell-cycle arrest in S cells, ↓ IC50, ↑ apoptosis in R, ↓ tumor volume, weight, pulmonary metastases in X		S: A549, H1299; R: A549/DDP; X: A549	[171]
				cells			S: A549; R: A549/DDP	[59]
62	miR-200b				↓ IC50, colony formation, ↑ apoptosis and cell-cycle arrest in G2/M phase in S, R, ↓ tumor volume in X	↑ IC50, colony formation	S: SPC-A1, A549; R: SPC-A1/DTX; X: SPC-A1/DTX	[158]
63	miR-200c				↓ cell viability, proliferation invasion, EMT ↑ apoptosis in S, ↓ liver metastasis*		S: H1299, H596, H522	[38]
64	miR-200bc/429 cluster	BCL2 and XIAP, anti-apoptotic	luciferase reporter assay, Western blot	cells	↓ IC50 VCR, CDDP, VP-16, and ADR, but not to 5-Fu, ↑ apoptosis	IC50	S: A549/CA549; R: A549/CA549/CDDP	[172]

65	miR-202	KRas	luciferase reporter assay, Western blot		↓ cell viability, IC50 ↑ apoptosis in S, ↓ tumor volume in X		S: NCI-H441, A549; X: A549	[32]
66	miR-202-5p	MDR1	luciferase assay, Western blot			↑ proliferation in S, R	S: A549; R: A549 /DDP	[173]
67	miR-203	DKK1	luciferase assay, qRT-PCR, Western blot	tissues	↓ IC50, cell proliferation, ↑ apoptosis, in S		S: A549, H460	[174]
68	miR-204	ZEB2	luciferase assay, Western blot			↑ IC50 in S	S: A549, SPCA-1	[175]
69		CAV1, AKT pathway	luciferase assay, qRT-PCR, Western blot		↓ IC50, ↑ mitochondrial apoptosis in R, ↓ tumor volume in X	↑ IC50 in S	S: A549, PC9 R: CR-A549, CR-PC9; X: CR-A549	[176]
70	miR-206	MET	luciferase assay, Western blot	tissues, cells	↓ cell viability, EMT, migration, invasion in R	↑ cell viability, EMT, migration and invasion in S	S: A549, H1299; R: A549/DDP, H1299/DDP	[177]
71	miR-214	c-Myc, CSCs, EMT	luciferase assay, Western blot		↓ CSCs (cisplatin-enriched CD133+, ALDH+ population, spheroid formation) in S	↑ β-catenin, Cyclin E, EZH2, Survivin in S	S: H460, H1299, A549	[178]
72	miR-216	Beclin-1, autophagy	luciferase assay, Western blot	cells	↑ apoptosis in R	↓ apoptosis in S	S: A549; R: A549 /DDP	[179]
73	miR-216b	c-Jun, c-Jun/Bcl-xl pathway, apoptosis			↓ IC50 in S ↓ tumor weight in X	↑ IC50 in S	S: A549, PC9; X: A549	[24]
74	miR-217	KRAS	luciferase assay, qRT-PCR, Western blot	cells	↓ cell viability in S	↑ cell viability in S	S: SPC-A-1, A549	[180]
75	miR-218	RUNX2, angiogenesis by enhancing endothelial	luciferase assay, Western blot	cells	↓ IC50, G0/S transition, cell invasion, MDR1, BCL-2, CyclinD1, ↑ apoptosis in R		S: A549; R: A549/DDP	[181]

		cell proliferation, invasion, and tube formation						
76	miR-219a-5p	FGF-9 proliferation	luciferase assay, qRT-PCR, Western blot, IHC staining	tissues, cells	↓ cell viability, proliferation, colony formation, ↑ cell-cycle arrest at G0/G1 in R, ↓ tumor volume, weight in X		S: A549, SPC-A1; R: A549-R, SPC-A1-R X: A549, A549-R	[182]
77	miR-224			cells			S: A549; R: A549/DDP	[118]
78	miR-203	DKK	luciferase assay, Western blot	tissues	↓ IC50, ↑ apoptosis in S, ↓ tumor weight in X		S: A549, H460; X: A549, H460	[174]
79	295-5p	STAT, EMT	luciferase reporter assay, Western blot			↓ inhibitory rate, ↑ IC50	S: A549, H1650; R: A549/DDP, H1650/DDP	[183]
80	miR-320b,d,e			cells			S: A549; R: A549/DDP	[59]
81	miR-324-3p	GPX4, ferroptosis	luciferase assay, qRT-PCR, Western blot	cells	↓ cell viability, colony formation, ↑ apoptosis in R, ↑ ferroptosis in S, R		S: A549; R: A549/DDP	[184]
82	miR-377-3p	GOT1, altered metabolism	luciferase reporter assay, Western blot	cells	↓ cell viability, ↑ apoptosis in R	↑ cell viability, ↓ apoptosis in S	S: A549, H1299, Calu-3; R: A549/DDP, H1299/DDP, Calu-3/DDP	[185]
83	miR-378	sCLU	luciferase reporter assay, Western blot	tissues, cells	↓ IC50, ↑ apoptosis in R, ↓ tumor volume in X		S: A549, Anip973; R: A549/cDDP, Anip973/cDDP; X: A549/cDDP	[78]
84	miR-379	EIF4G2, the recruitment of mRNA during	luciferase reporter assay, Western blot	tissues, cells	↓ proliferation, colony formation, ↑ apoptosis in R, ↓ tumor volume in RX	↑ cell proliferation, colony formation, ↓	H1299 and A549, R6 A549/r X: A549, R6 A549/r	[79]

		translational initiation				apoptosis in S, ↑ tumor volume in SX		
85	miR-381	activation of nuclear factor (NF)-κB through repression of inhibitor of differentiation 1 (ID1)	luciferase reporter assay, qRT-PCR, Western blot	cells	↓ cell viability, colony formation, ↑ cell-cycle arrest at G0/G1 phase in S, ↓ cell viability, ↑ apoptosis in R, ↓ tumor volume in X		S: A549, NCI-H460; R: A549/DPP	[80]
86	miR-448	siRNA SATB1	luciferase reporter assay, qRT-PCR, Western blot	tissues, cells	↓ IC50, proliferation in R, ↑ cell-cycle arrest at G0/G1 phase, apoptosis in R, S	↑ IC50 in R	S: A549; R: A549/DPP	[186]
87	miR-451a	MCL-1	luciferase reporter assay, qRT-PCR, Western blot	Cells	↓ IC50 in R, ↓ tumor volume in X		S: A549; R: A549/DPP; X: A549	[81]
88	miR-451	TRIM66	luciferase reporter assay, qRT-PCR, Western blot		↓ proliferation, ↑ apoptosis in S, R		S: A549, H157; R: A549/DDP, H157/DDP	[82]
89					↓ viability, colony number, ↑ apoptosis in S, ↓ tumor volume in X		S: A549; X: A549	[83]
90	miR-454	Stat3	luciferase reporter assay, Western blot	cells	↓ IC50, ↑ apoptosis	↑ IC50, ↓ apoptosis	S: A549, H157;	[187]
91	miR-485	CD44	luciferase reporter assay, qRT-PCR, Western blot	cells	↓ stemness in R cells		S: A549; R: A549/DDP	[188]
92	miR-486-5p	TWF1, EMT	luciferase reporter assay, qRT-PCR, Western blot, Immunohistochemistry	cells	↓ IC50 in S, R, ↓ tumor weight in X	↑ IC50 in S, R	S: A549; R: A549/DDP; X: A549/DDP	[84]
93	miR-493	TCRP1 apoptosis			↓ growth, ↑ apoptosis in S, R		S: A549 cells R: A549/DDP cells	[189]

94	miR-495	ATP7A, drug transportation Drug efflux	luciferase reporter assay, qRT-PCR, Western blot	tissues, cells	↓ cell viability, intracellular DDP accumulation in S, R	↑ cell viability, in S, R	S: A549;<br>R: A549/DDP	[40]
95	miR-497	BCL2 anti-apoptotic	luciferase reporter assay, Western blot	cells	↓ cell viability VCR, ↑ apoptosis in R	↑ cell viability in S	S: A549;<br>R: A549/DDP	[41]
96	miR-497-5p	mTOR, apoptosis	luciferase reporter assay, Western blot		↓ cell viability, IC50 in S	↑ cell viability, IC50, ↓ apoptosis in S	S: A549, SK-MES-1	[190]
97	miR-503-3p	BCL2 anti-apoptotic	luciferase reporter assay	cells	↓ cell viability ↑ apoptosis in R	↑ cell viability in S	S: A549;<br>R: A549/CDDP	[191]
98	miR-503				↓ IC50, ↑ apoptosis in S, R	↑ IC50, ↓ apoptosis in S, R	S: A549;<br>R: A549/CDDP	[192]
99	miR-513a-3p	GSTP1	luciferase reporter assay, Western blot	cells	↓ IC50 in R	= IC50 in A549	S: A549, SPCA-1<br>R: A549/CDDP SPCA-1	[193]
100	miR-516b-5p	STAT3glycolysis	luciferase reporter assay, qRT-PCR, Western blot		↓ cell viability, glucose consumption and lactic acid formation in S,<br>↓ tumor volume, weight in X		S: A549, H1299;<br>X: A549	[194]
101	miR-539	DCLK1	luciferase reporter assay, qRT-PCR,	Cells, tissues	↓ cell viability, IC50, cell-cycle arrest in G0/G1, invasion, migration, ↑ apoptosis in R	↑ cell viability, IC50, cell-cycle arrest in G0/G1, ↓ apoptosis in R	S: A549, H1299;<br>R: A549/DDP, H1299/DDP	[195]
102	miR-556-3p	AK4, EMT under hypoxia	luciferase reporter assay, qRT-PCR, Western blot			↑ proliferation, colony formation, migration, ↓ apoptosis		[170]
103	miR-608	TEAD2, Hippo-yes-associated protein signaling pathway	luciferase reporter assay, qRT-PCR, Western blot		↓ cell viability, ↑ apoptosis	↑ cell viability	A549	[196]

104	miR-613	GJA1, TBP and EIF-4E	Western blot	cells	↓ cell viability,<br>exo-miRs: ↓ cell viability, migration ↑ apoptosis, DNA damage in S, R<br>exo-miRs: ↓ tumor volume, weight in X		S: A549;<br>R: A549/DDP;<br>X: A549/DDP	[85]
105	miR-627-5	GJA1	luciferase reporter assay, Western blot		↓ proliferation, migration, colony formation, ↑ apoptosis in S, ↓ tumor volume, weight, Ki67 in X		S: H1299, A549;<br>X: A549	[86]
				cell exosomes			S: A549	[27]
106	miR-647	IGF						[197]
107	miR-886			cells			S: A549;<br>R: A549/DDP	[118]
108	miR-1236-3p	TPT1	luciferase reporter assay, qRT-PCR, Western blot	cells	↓ proliferation, ↑ apoptosis in S, R		S: A549;<br>R: A549/DDP	[198]
109	miR-1244	Bax, MEF2D, cyclin D1 and p53	qRT-PCR, Western blot		↓ proliferation, ↑ apoptosis in S		S: A549, H522	[33]
110	miR-1308			cells			S: A549;<br>R: A549/DDP	[59]
111	miR-2861			cells			S: A549;<br>R: A549/DDP	[59]
112	miR-1273a	SDCBP (MDA-9), metastasis	luciferase reporter assay, qRT-PCR, Western blot	cell exosomes, plasma exosomes	↓ cell viability, ↑ apoptosis		A549	[27]

DDP–cisplatin; DDP-S miRNA–miRNA associated with DDP sensitivity; R–chemotherapy-resistant cell line; S–chemotherapy-sensitive cell line; X–mice xenograft based on LC cell lines.
